# Abdominal negative-pressure therapy: a new method in countering abdominal compartment and peritonitis - prospective study and critical review of literature

**DOI:** 10.1186/2110-5820-2-S1-S23

**Published:** 2012-12-20

**Authors:** Haralds Plaudis, Agris Rudzats, Liene Melberga, Ita Kazaka, Olegs Suba, Guntars Pupelis

**Affiliations:** 1 Department of General and Emergency Surgery, Riga East Clinical University Hospital "Gailezers", 2 Hipokrata Street, Riga, LV 1038, Latvia; 2 Intensive Care Clinic, Riga East Clinical University Hospital "Gailezers", 2 Hipokrata Street, Riga, LV 1038, Latvia

## Abstract

**Background:**

Application of abdominal negative-pressure therapy (NPT) is lifesaving when conservative measures fail to reduce sustained increase of the intra-abdominal pressure and it is impossible to achieve source control in a single operation due to severe peritonitis. The aim of this study is to share the initial experience with abdominal NPT in Latvia and provide a review of the relevant literature.

**Methods:**

In total, 22 patients were included. All patients were treated with KCI^® ^ABThera™ NPT systems. Acute Physiology and Chronic Health Evaluation II (APACHE II) score on admission, daily sequential organ failure assessment score and Mannheim peritonitis index (MPI) were calculated for severity definition. The frequency of NPT system changes, daily amount of aspirated fluid effluent and the time of abdominal closure were assessed. The overall hospital and ICU stay, as well as the outcomes and the complication rate, were analysed.

**Results:**

A complicated intra-abdominal infection was treated in 18 patients. Abdominal compartment syndrome due to severe acute pancreatitis (SAP), secondary ileus and damage control in polytrauma were indications for NPT in four patients. The median age of the patients was 59 years (range, 28 to 81), median APACHE II score was 15 points (range, 9 to 32) and median MPI was 28 points (range, 21 to 40), indicating a prognostic mortality risk of 60%. Sepsis developed in all patients, and in 20 of them, it was severe. NPT systems were changed on a median of every 4 days, and abdominal closure was feasible on the seventh postoperative day without needing a repeated laparotomy. Two NPT systems were removed due to bleeding from the retroperitoneal space in patients with SAP. Intestinal fistulae developed in three patients that were successfully treated conservatively. Incisional hernia occurred in three patients. The overall ICU and hospital stay were 14 (range, 5 to 56) and 25 days (range, 10 to 87), respectively. Only one patient died, contributing to the overall mortality of 4.5%.

**Conclusions:**

Application of abdominal NPT could be a very promising technique for the control of sustained intra-abdominal hypertension and management of severe sepsis due to purulent peritonitis. Further trials are justified for a detailed evaluation of abdominal NPT indications.

## Background

Management of the open abdomen (OA) is a challenging task and is associated with a number of complications including persistent sepsis, high entero-atmospheric fistula rate, development of multiple organ dysfunction and frozen abdomen [1-3]. Abdominal negative-pressure therapy (NPT) has opened new opportunities in the treatment of patients with intra-abdominal hypertension (IAH), complicated intra-abdominal infection (IAI) and severely polytraumatised patients in whom damage control laparotomy is mandatory [1]. During the latest decades, a number of techniques have been described on the management of patients with OA including the Bogota bag, towel clamp and zipper technique; however, results of the treatment have not been satisfactory [2]. Implementation of the Wittmann patch and home-made NPT systems gave stimulus to the development of commercially available systems that are at present considered to be the most effective [3]. Nevertheless, clinical experience in the application of different abdominal NPT techniques is scarce and lacks definitive opinion about the indications and timing of NPT [1,2]. This method is still associated with high morbidity and high incidence of ventral hernia formation in surviving patients caused by difficulties in definitive closure of the abdominal wall after prolonged application of NPT [1,4,5]. This study uses the results of having implemented the principle of topical negative-pressure therapy in general surgery since 2007. The aim of the study is to describe the initial experience with NPT in the management of patients with abdominal compartment syndrome (ACS) and severe peritonitis as well as provide a review of recent literature regarding the application of the topical negative-pressure therapy in the management of patients with an open abdomen.

## Methods

In total, 25 surgical patients underwent abdominal NPT during the period from 2007 to 2012. In three patients, home-made systems were used. Since 2010, vacuum-assisted abdominal closure systems ABThera™ (KCI^® ^Riga, Latvia) were applied to the 22 patients who were prospectively included in the study. The indication for NPT was complicated intra-abdominal infection with severe sepsis due to total purulent peritonitis and/or ACS. The decision to apply NPT in cases of advanced peritonitis was based on evidence that complete source control would require a repeated laparotomy. The indication for decompressive laparotomy was a sustained increase of intra-abdominal pressure (IAP) >25 mmHg for more than 24 h despite aggressive complex conservative therapy including continuous veno-venous haemofiltration and percutaneous drainage of the intra-abdominal fluid collections if indicated [6]. During the application of the NPT systems, all patients were treated in the ICU. Before the operation, the patient's condition and severity of sepsis were assessed by calculation of the Acute Physiology and Chronic Health Evaluation II (APACHE II) score on admission and assessment of organ dysfunction according to the Sequential Organ Failure Assessment (SOFA) score [7]. After the operation, the Mannheim peritonitis index (MPI) was calculated to predict the individual risk of death due to peritonitis [8]. Dynamic assessment of organ dysfunction and clinical course of sepsis was done by daily calculation of the SOFA score. IAP was measured with the patient in supine position, instillation of 20 mL of sterile saline solution and the linea axillaris media and crista iliaca cross point as the zero point. IAP was measured twice daily during the patients' ICU stay. IAH was defined by a sustained or repeated pathological elevation in IAP ≥12 mmHg. ACS was diagnosed when sustained increase of the IAP >20 mmHg and one new organ dysfunction were detected [9]. Abdominal perfusion pressure (APP) was calculated in all patients with IAH and defined as the mean arterial pressure (MAP) minus the IAP. The decision to discontinue negative-pressure therapy and to perform permanent closure of the abdominal cavity was based on preoperative evidence of stable regression of the inflammatory response, regression of organ dysfunction and sepsis. Intraoperative evidence of a visually clean abdomen (absence of abscesses or purulent exudate, disappearance of fibrin) and well-perfused bowels with recovered transit determined the decision to close the abdomen. Bacteriological samples from the abdominal cavity were taken during all operations; however, we did not wait until all intraoperative cultures would become negative. Blood cultures from the abdominal effluent were collected until regression of sepsis and stable decrease of the C-reactive protein (CRP) in the blood were achieved. Positive blood culture was defined as septicaemia. The complication rate, hospital and ICU stay and main outcomes were analysed for all patients. Application of NPT was approved by the institutional ethics committee. Informed consent was obtained according to institutional practice. Statistical data analysis was done using SPSS version 17.0. All data have been changed to median values and range.

## Results

Starting from the year 2010, 22 patients were treated with commercially available abdominal NPT systems and prospectively included in study. The proportion of males and females in the population of the study was 3:1. Patient median age was 59 years (range, 21 to 81). Initially, NPT was applied in patients with severe acute pancreatitis who develop ACS. The next step was application of NPT to patients with severe peritonitis and to polytraumatised patients. The main indications for abdominal NPT are summarised in Table 1. Disease severity assessment before application of NPT revealed that the median APACHE II score at admission was 15 points (range, 9 to 32); following the intervention, the median MPI in patients with peritonitis reached 28 points (range, 21 to 40). In nine patients, septic shock aggravated the clinical course, and prolonged mechanical ventilatory support was necessary in 14 patients (Table 2). In all patients, the median of 2 (range, 1 to 6) dressing changes were done in the time interval of 4 days (range, 1 to 5). Complete fascial closure was achieved in the median of 7 days (range, 4 to 18) after the first application of NPT. No repeated operations were necessary after removal of the NPT systems, and permanent abdominal closure was possible in all patients. Abdominal NPT facilitated normalisation of the IAP from 20.5 mmHg (range, 18 to 28) before the therapy to the median of 13.5 mmHg (range, 11 to 17) at the point when permanent closure was possible, and fascial closure was completed in all patients who suffered ACS. Before the application of NPT, the median MAP was 80 mmHg (range, 69 to 97), and it reached 88 mmHg (range, 79 to 107) after fascial closure. Improvement of the splanchnic perfusion was reflected in the increase of the APP from 75 mmHg (range, 52 to 82) to 78 mmHg (range, 69 to 87). The application of commercially available NPT systems in our patients allowed obtaining the median of 575 mL (range, 400 to 1,850) of liquid effluent from the abdominal cavity in a 24-h period. Bleeding from the retroperitoneal tissue occurred in two patients (9%) with SAP, and it was the reason for discontinuation of NPT. In three patients (13.6%), small-bowel fistulae developed after the first NPT system change. Fistulae were closed primarily with sutures, and NPT was continued. Development of entero-atmospheric fistulae was not observed during the next NPT system change. Regression of sepsis was stable in all patients despite the fact that positive blood cultures proved septicaemia in six patients (27.2%) with severe sepsis. Bacteriological samples from the abdominal cavity revealed mainly Gram-negative bacteria (*Klebsiella oxytoca *(two), *Clostridium clostridiforme *(one), *Bacteroides fragilis *(two), *Enterococcus faecalis *(four), *K. oxytoca *(ESBL) (one), *Escherichia coli *(two), *Enterobacter cloaceae *(two), *Streptococcus anginosus *(one) and *Prevotella buccae *(two)), and blood cultures revealed mainly Gram-positive bacteria (*Staphylococcus epidermidis *(two), *Staphylococcus hominis *(one), *B. fragilis *(one), *Bacteroides uniformis *(one) and *Citrobacter freundii *(one)).

**Table 1:** Indications for abdominal NPT

Clinical situation	Number
Abdominal compartment syndrome	6^a^
Purulent peritonitis with severe sepsis	16
Polytraumatised patients	1

^a^One patient with peritonitis had ACS.

**Table 2:** Severity of the disease and main complications

Parameter	Value
APACHE II (points)	15 (range, 9 to 32)
Mannheim peritonitis index (points)	28 (range, 21 to 40)
SOFA score (points)	8 (range, 3 to 13)
Use of vasopressors (number)	9
Use of mechanical ventilation (number)	14

**Figure 1:**
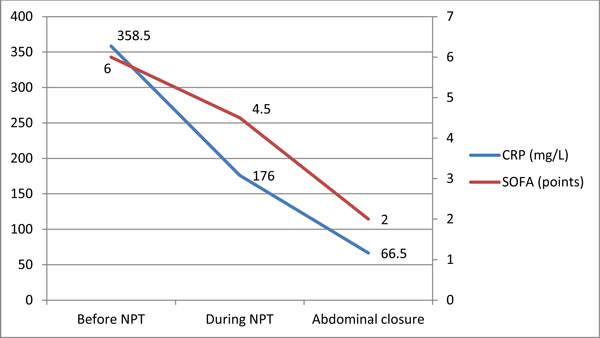
**Dynamics of CRP and regression of MODS during abdominal NPT**.

Abdominal NPT was associated with a fast decrease of plasma CRP levels, which dropped to the median of 83 mg/L (range, 28 to 257) at the point of the abdominal closure. Dynamics of the CRP and regression of multiple organ failure syndrome (MODS) are shown in Figure 1. Median ICU stay in our study population was 14 days (range, 5 to 56), and hospital stay was 25 days (range, 10 to 87). Only one patient (4.5%) with ACS died in our series.

## Discussion

### General

For decades, surgery has been the cornerstone of abdominal sepsis treatment. Elimination of the septic focus, removal of necrotic tissue and drainage of purulent material in a single operation is every surgeon's desire; however, reaching all these goals is not always feasible. Despite advances in surgery, antibacterial therapy and intensive care support, mortality and morbidity remain high [10].

Management of the OA in cases of ACS or advanced peritonitis has been considerably improved by implementation of the NPT, especially considering treatment of complicated non-trauma patients [11]. More than a decade has passed since NPT was introduced as a method for the management of patients with OA. NPT has emerged from its implication in the therapy of patients with chronic wounds to its application even in cardiothoracic surgery [12,13].

### Inflammatory fluid, cytokines, gut oedema and MODS

More than 70% of all immune cells are localised in the gastrointestinal tract, attributing the gut as a cytokine-generating organ [14,15] and an important mediator of inflammation [16], the so-called brain that drives the immune response. Pathophysiological mechanisms associated with the inflammatory response are leading to capillary leakage, IAH and development of multiple organ failure [17-19]. The local peritoneal inflammatory cascade is known to be similar to the systemic inflammatory response [20].

Peritoneal fluid is a plasma ultrafiltrate and may be considered as a representative of the biologic profile of plasma. The mesenteric lymphatic and venous system play a key role in removing interstitial fluid of the gut. By causing gut oedema, inflammation results in the disturbances of the venous outflow that can lead to elevated venous pressures and transvascular fluid flux that exceeds greatly the draining capacity of the abdominal and the thoracic lymphatic system. Under these circumstances, inflammatory fluid is retained in the gut wall, intestinal villi secrete fluid into the gut lumen, and the luminal fluid does not rapidly regress from the gastrointestinal tract, contributing to IAH and even ACS [14,21]. Animal and human studies show that circulation of lymph containing a high concentration of cytokines can cause severe liver, kidney and lung injury and the development of MODS and is therefore associated with poor outcomes [16,18,20-22]. In animal models, abdominal NPT was shown to prevent the development of lung injury and was associated with decreases in leukocytes migrated to the kidney and liver [16,18]. These results clearly show that extensive removal of mediator-rich peritoneal fluid may decrease the pro-inflammatory characteristics of abdominal exudate and thus improve outcomes [18].

### Indications and treatment

Balance between the pro- and anti-inflammatory responses can be achieved by early intensive therapy where definition of adequate fluid replacement strategy is crucial [18]. Severely ill patients that are heavily resuscitated are at very high risk of the development of bowel oedema and ACS. It is well known that temporary abdominal closure (TAC) devices provide the augmented drainage of mediator-rich peritoneal fluid contributing to systemic and locoregional inflammatory response [18,21].

Techniques for TAC are various and have evolved from the basic methods, with home-made systems that can be used to simply contain the visceral contents, to more dynamic modern devices, such as the commercially available NPT systems for active abdominal therapy. More than a decade has passed since NPT was introduced as a method for the management of patients with OA [10,12,13]. Compared to other techniques, NPT systems are intended to provide active abdominal therapy by controlling the abdominal contents and actively removing exudate from all the abdominal compartments [4,18,21,23]. The level of vacuum can be altered individually, although the best results have been demonstrated with a pressure of 125 mmHg [2,10]. The recommended interval between dressing changes is usually 24 to 72 h; however, the actual timing of dressing changes may vary individually depending on the patient's clinical status and indications [1,2,10,24].

Nowadays, abdominal NPT is indicated in patients with severe abdominal infection in cases when the source of sepsis cannot be fully controlled, in cases of ACS or in patients with acute mesenteric ischemia that can be considered for a second look [10,24-26]. Patients in whom damage control laparotomy is indicated should be considered for the application of NPT to avoid the development of ACS, visceral hypoperfusion and multiple organ failure [9,10,24,26-29]. Unfortunately, until now, randomised trials demonstrating the superiority of any particular TAC technique are lacking [5,30].

### Abdominal closure and complications

The success rate of abdominal closure mostly depends on the aetiology of the open abdomen [31]. Currently, application of abdominal NPT has extended the 'window' for delayed primary fascial closure from 4 to 5 days (in cases of trauma when principles of 'damage control' are applied) to as long as 1 month (in patients with complicated IAI) after the initial procedure [28,32]. A study by Kaplan et al. [5] clearly shows that, if the abdomen is not primarily closed within 7 to 10 days, adhesion formation and fascia retraction can render this impossible. If the application time is too long and the NPT system changes are too frequent, the rate of entero-cutaneous fistula formation, bowel obstruction, bleeding and other complications can increase [26,27,32-34]. Mortality rates vary between 7% and 38% [4,35] and can even reach 50% in patients with peritonitis, severe sepsis or septic shock [10,25].

Different conservative and surgical methods have been described to overcome these problems, including component separation techniques, split-thickness skin grafting, use of biological mesh materials, etc. [11,27,29,36,37]. However, indications for appropriate fascial closure time are still lacking, and in the late stage of the disease, surgeons are mostly fighting with complications that are associated with NPT application [3,24]. Results of this study demonstrate certain evidence that clinical assessment of the abdominal cavity considering clearance of the purulent contents and fibrin deposits and signs of well-perfused functioning bowels may indicate that definitive abdominal closure is possible.

## Conclusions

Abdominal NPT could be a highly promising method in the management of patients with increased IAP and severe sepsis due to purulent peritonitis. Regression of sepsis, normalisation of CRP and visual confirmation of complete source control may help to select patients for definitive abdominal closure following NPT even in cases when abdominal bacterial cultures remain positive. Further trials should study indications for abdominal NPT in detail.

## Key message

• Regression of sepsis, normalisation of CRP and visual confirmation of complete source control may help to select patients for definitive abdominal closure after NPT even in cases when abdominal bacterial cultures are positive.

## List of abbreviations used

ACS: abdominal compartment syndrome; APP: abdominal perfusion pressure; CRP: C-reactive protein; IAH: intra-abdominal hypertension; IAP: intra-abdominal pressure; ICU: intensive care unit; MAP: mean arterial pressure; MPI: Manheim peritonitis index; MODS: multiple organ failure syndrome; NPT: negative-pressure therapy; OA: open abdomen; SAP: severe acute pancreatitis

## Competing interests

The authors declare that they have no competing interests.

## Authors' contributions

HP, GP, and IK contributed to conceiving the study, interpreting the results, drafting and revising the manuscript, and approving the manuscript in its final form. AR and OS contributed to the treatment of the patients and the acquisition and management of the data. HP, LM and IK contributed to the statistical analysis and data acquisition. LM contributed to the data acquisition and in providing data input in the electronic database.

## References

[B1] Boele van Hensbroek P Wind J Dijkgraaf MG Busch OR Carel Goslings J Temporary closure of the open abdomen: a systematic review on delayed primary fascial closure in patients with an open abdomen World J Surg 2009 33 199 207 10.1007/s00268-008-9867-3 19089494 PMC3259401

[B2] Haldipur N Cooper B Sanyal S Managing the open abdomen J R Army Med Corps 2006 152 143 147 17295011 10.1136/jramc-152-03-06

[B3] Björck M Bruhin A Cheatham M Hinch D Kaplan M Manca G Wild T Windsor A Classification, an important step to improve the management of patients with an open abdomen World J Surg 2009 33 1154 1157 10.1007/s00268-009-9996-3 19373507

[B4] Batacchi S Matano S Nella A Zagli G Bonizzoli M Pasquini A Anichini V Tucci V Manca G Ban K Valeri A Peris A Vacuum-assisted closure device enhances recovery of critically ill patients following emergency surgical procedures Crit Care 2009 13 194 202 10.1186/cc8033 19961614 PMC2811940

[B5] Kaplan M Banwell P Orgill DP Ivatury RR Moore FA Miller P Nicholas J Henry S Guidelines for the management of the open abdomen Wounds 2005 17 1 24

[B6] Pupelis G Plaudis H Grigane A Zeiza K Purmalis G Continuous veno-venous haemofiltration in the treatment of severe acute pancreatitis: 6-year experience HPB (Oxford) 2007 9 295 301 10.1080/13651820701329225 18345308 PMC2215400

[B7] Vincent JL de Mendonca A Cantraine F Moreno R Takala J Suter PM Sprung CL Colardyn F Blecher S Use of the SOFA score to assess the incidence of organ dysfunction/failure in intensive care units: results of a multicenter, prospective study Crit Care Med 1998 26 1793 1800 10.1097/00003246-199811000-00016 9824069

[B8] Wacha H Linder MM Feldmann U Wesch G Gundlach E Steifensand RA Mannheim peritonitis index--prediction of risk of death from peritonitis Theoretical Surg 1987 1 169 177

[B9] Malbrain ML Cheatham ML Kirkpatrick A Sugrue M Parr M De Waele J Balogh Z Leppäniemi A Olvera C Ivatury R D'Amours S Wendon J Hillman K Johansson K Kolkman K Wilmer A Results from the international conference of experts on intra-abdominal hypertension and abdominal compartment syndrome. I. Definitions Intensive Care Med 2006 32 1722 1732 10.1007/s00134-006-0349-5 16967294

[B10] Amin AI Shaikh IA Topical negative pressure in managing severe peritonitis: a positive contribution? World J Gastroenterol 2009 15 Suppl 27 3394 3397 19610140 10.3748/wjg.15.3394 PMC2712900

[B11] Kritayakirana K Maggio PM Brundage S Purtill MA Staudenmayer K Spain DA Outcomes and complications of open abdomen technique for managing non-trauma patients J Emerg Trauma Shock 2010 3 118 122 10.4103/0974-2700.62106 20606786 PMC2884440

[B12] Preston G An overview of topical negative pressure therapy in wound care Nurs Stand 2008 23 62 64 18988585 10.7748/ns2008.10.23.7.62.c6713

[B13] Eyileten Z Akar AR Eryilmaz S Sirlak M Yazicioglu L Durdu S Uysalel A Ozyurda U Vacuum-assisted closure and bilateral pectoralis muscle flaps for different stages of mediastinitis after cardiac surgery Surg Today 2009 39 947 954 10.1007/s00595-008-3982-5 19882316

[B14] Radhakrishnan RS Xue H Weisbrodt N Moore FA Allen SJ Laine GA Cox CS Resuscitation-induced intestinal edema decreases the stiffness and residual stress of the intestine Shock 2005 24 165 170 10.1097/01.shk.0000168873.45283.4c 16044088

[B15] Rezende-Neto JB Moore EE Masuno T Moore PK Johnson JL Sheppard FR Cunha-Melo JR Silliman CC The abdominal compartment syndrome as a second insult during systemic neutrophil priming provokes multiple organ injury Shock 2003 20 303 308 10.1097/01.shk.0000082487.34705.d3 14501942

[B16] Shah SK Jimenez F Walker PA Xue H Feeley TD Uray KS Norbury KC Stewart RH Laine GA Cox CS Peritoneal fluid: a potential mechanism of systemic neutrophil priming in experimental intra-abdominal sepsis Am J Surg 2012 203 211 216 10.1016/j.amjsurg.2010.12.012 21679918 PMC3177024

[B17] Richard SH Irene EK The pathophysiology and treatment of sepsis N Engl J Med 2003 348 138 150 10.1056/NEJMra021333 12519925

[B18] Kubiak BD Albert SP Gatto LA Snyder KP Maier KG Vieau CJ Roy S Nieman GF Peritoneal negative pressure therapy prevents multiple organ injury in a chronic porcine sepsis and ischemia/reperfusion model Shock 2010 34 525 534 10.1097/SHK.0b013e3181e14cd2 20823698

[B19] Benninger E Labler L Seifert B Trentz O Menger MD Meier C Moore-Olufemi SD Xue H Allen SJ Moore FA Stewart RH Laine GA Cox CS *In vitro *comparison of intra-effects of primary and secondary intra-abdominal hypertension on mesenteric lymph flow: implications for the abdominal compartment syndrome J Surg Res 2008 144 102 106 10.1016/j.jss.2007.02.021 17764694

[B20] Bokesch PM Kapural MB Mossad EB Cavaglia M Appachi E Drummond-Webb JJ Mee RBB Do peritoneal catheters remove pro-inflammatory cytokines after cardiopulmonary bypass in neonates? Ann Thorac Surg 2000 70 639 643 10.1016/S0003-4975(00)01453-3 10969693

[B21] Moore-Olufemi SD Xue H Allen SJ Moore FA Stewart RH Laine GA Cox CS Effects of primary and secondary intra-abdominal hypertension on mesenteric lymph flow: implications for the abdominal compartment syndrome Shock 2005 23 571 575 15897812

[B22] Kowal-Vern A Ortegel J Bourdon P Chakrin A Latenser BA Kimball D Casey LC Elevated cytokine levels in peritoneal fluid from burned patients with intra-abdominal hypertension and abdominal compartment syndrome Burns 2006 32 563 569 10.1016/j.burns.2005.12.010 16766124

[B23] Garner GB Ware DN Cocanour CS Duke JH McKinley BA Kozar RA Moore FA Vacuum-assisted wound closure provides early fascial reapproximation in trauma patients with open abdomens Am J Surg 2001 182 630 638 10.1016/S0002-9610(01)00786-3 11839329

[B24] Horwood J Akabar F Maw A Initial experience of laparostomy with immediate vacuum therapy in patients with severe peritonitis Ann R Coll Surg Engl 2009 91 681 687 10.1308/003588409X12486167520993 19785944 PMC2966252

[B25] Sartelli M A focus on intra-abdominal infections World J Emerg Surg 2010 5 Suppl 9 1 20 20302628 10.1186/1749-7922-5-9 PMC2848006

[B26] Schmelzle M Alldinger I Matthaei H Aydin F Wallert I Eisenberger CF Schulte E Dizdar L Topp SA Yang Q Knoefel WT Long-term vacuum-assisted closure in open abdomen due to secondary peritonitis: a retrospective evaluation of a selected group of patients Dig Surg 2010 27 Suppl 4 272 278 20664203 10.1159/000314609

[B27] Keramati M Srivastava A Sakabu S Rumbolo P Smock M Pollack J Troop B The Wittmann patch as a temporary abdominal closure device after decompressive celiotomy for abdominal compartment syndrome following burn Burns 2008 34 493 497 10.1016/j.burns.2007.06.024 17949916

[B28] Petersson U Acosta S Björck M Vacuum-assisted wound closure and mesh-mediated fascial traction--a novel technique for late closure of the open abdomen World J Surg 2007 31 2133 2137 10.1007/s00268-007-9222-0 17879112

[B29] Cheatham ML Malbrain MLNG Kirkpatrick A Sugrue M Parr M De Waele J Balogh Z Leppäniemi A Olvera C Ivatury R D'Amours S Wendon J Hillman K Wilmer A Results from international conference of experts on intra-abdominal hypertension and abdominal compartment syndrome. II. Recommendations Int Care Med 2007 33 951 962 10.1007/s00134-007-0592-4 17377769

[B30] Shah SK Jimenez F Letourneau PA Walker PA Moore-Olufemi SD Stewart RH Laine GA Cox CS Strategies for modulating the inflammatory response after decompression from abdominal compartment syndrome Scand J Trauma Resusc Emerg Med 2012 20 25 10.1186/1757-7241-20-25 22472164 PMC3352320

[B31] Benninger E Labler L Seifert B Trentz O Menger MD Meier C Abdominal hypertension development after different temporary abdominal closure techniques J Surg Res 2008 144 102 106 10.1016/j.jss.2007.02.021 17764694

[B32] Verdam FJ Dolmans DE Loos MJ Raber MH de Wit RJ Charbon JA Vroemen JP Delayed primary closure of the septic open abdomen with a dynamic closure system World J Surg 2011 35 2348 2355 10.1007/s00268-011-1210-8 21850603 PMC3170463

[B33] Barker DE Green JM Maxwell RA Smith PW Mejia VA Dart BW Cofer JB Roe SM Burns RP Experience with vacuum-pack temporary abdominal wound closure in 258 trauma and general and vascular surgical patients J Am Coll Surg 2007 204 784 792 10.1016/j.jamcollsurg.2006.12.039 17481484

[B34] Hlebowicz J Hansson J Lindstedt S Microvascular blood flow response in the intestinal wall and the omentum during negative wound pressure therapy of the open abdomen Int J Colorectal Dis 2012 27 397 403 10.1007/s00384-011-1317-2 21938450 PMC3281201

[B35] Barker DE Kaufman HJ Smith LA Ciraulo DL Richart CL Burns RP Vacuum pack technique of temporary abdominal closure: a 7-year experience with 112 patients J Trauma 2000 48 201 206 10.1097/00005373-200002000-00001 10697075

[B36] Shaikh IA Ballard-Wilson A Yalamarthi S Amin AI Use of topical negative pressure in assisted abdominal closure does not lead to high incidence of enteric fistulae Colorectal Dis 2010 12 Suppl 9 931 934 19438884 10.1111/j.1463-1318.2009.01929.x

[B37] Ivatury RR Update on open abdomen management: achievements and challenges World J Surg 2009 33 1150 1153 10.1007/s00268-009-0005-7 19350323

